# 2-[(Pyridin-3-yl­amino)­meth­yl]phenol

**DOI:** 10.1107/S160053681104685X

**Published:** 2011-11-12

**Authors:** Jing Xu, Shan Gao, Seik Weng Ng

**Affiliations:** a Key Laboratory of Functional Inorganic Material Chemistry, Ministry of Education, Heilongjiang University, Harbin 150080, People’s Republic of China; b Department of Chemistry, University of Malaya, 50603 Kuala Lumpur, Malaysia; c Chemistry Department, Faculty of Science, King Abdulaziz University, PO Box 80203 Jeddah, Saudi Arabia

## Abstract

In the title compound, C_12_H_12_N_2_O, the aromatic rings at either ends of the –CH_2_–NH– link are twisted by 68.79 (7)°. In the crystal, the hy­droxy substituent is a hydrogen-bond donor to the N atom of the pyridine ring of an adjacent mol­ecule, and the hydrogen bond generates a chain along the *b* axis; it is also a hydrogen-bond acceptor to the amino group of another adjacent mol­ecule. The two hydrogen bonds lead to the formation of a layer structure.

## Related literature

For the *N*-salicyl­idene-3-amino­pyridine precursor, see: Csaszar (1990 ▶); Kaya & Guelel (2005 ▶); Robert *et al.* (2009 ▶). For a related structure, see: Xu *et al.* (2011 ▶).
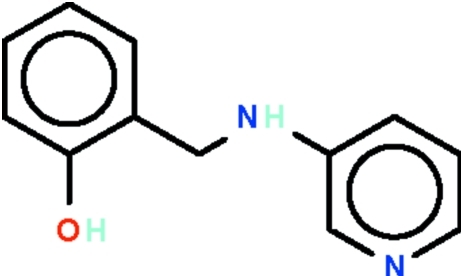


## Experimental

### 

#### Crystal data

C_12_H_12_N_2_O
*M* *_r_* = 200.24
Monoclinic, 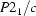

*a* = 5.8386 (11) Å
*b* = 13.399 (3) Å
*c* = 13.169 (3) Å
β = 90.519 (6)°
*V* = 1030.1 (4) Å^3^
*Z* = 4
Mo *K*α radiation
μ = 0.08 mm^−1^
*T* = 293 K
0.21 × 0.12 × 0.12 mm

#### Data collection

Rigaku R-AXIS RAPID IP diffractometer
Absorption correction: multi-scan (*ABSCOR*; Higashi, 1995 ▶) *T*_min_ = 0.983, *T*_max_ = 0.990
9845 measured reflections
2358 independent reflections
1879 reflections with *I* > 2σ(*I*)
*R*_int_ = 0.042

#### Refinement

*R*[*F* ^2^ > 2σ(*F* ^2^)] = 0.047
*wR*(*F* ^2^) = 0.137
*S* = 1.08
2358 reflections
144 parameters
2 restraints
H atoms treated by a mixture of independent and constrained refinement
Δρ_max_ = 0.26 e Å^−3^
Δρ_min_ = −0.18 e Å^−3^

### 

Data collection: *RAPID-AUTO* (Rigaku, 1998 ▶); cell refinement: *RAPID-AUTO*; data reduction: *CrystalClear* (Rigaku/MSC, 2002 ▶); program(s) used to solve structure: *SHELXS97* (Sheldrick, 2008 ▶); program(s) used to refine structure: *SHELXL97* (Sheldrick, 2008 ▶); molecular graphics: *X-SEED* (Barbour, 2001 ▶); software used to prepare material for publication: *publCIF* (Westrip, 2010 ▶).

## Supplementary Material

Crystal structure: contains datablock(s) global, I. DOI: 10.1107/S160053681104685X/xu5384sup1.cif

Structure factors: contains datablock(s) I. DOI: 10.1107/S160053681104685X/xu5384Isup2.hkl

Supplementary material file. DOI: 10.1107/S160053681104685X/xu5384Isup3.cml

Additional supplementary materials:  crystallographic information; 3D view; checkCIF report

## Figures and Tables

**Table 1:** Hydrogen-bond geometry (Å, °)

*D*—H⋯*A*	*D*—H	H⋯*A*	*D*⋯*A*	*D*—H⋯*A*
O1—H1o⋯N2^i^	0.86 (1)	1.80 (1)	2.6568 (16)	175 (2)
N1—H1n⋯O1^ii^	0.88 (1)	2.38 (1)	3.2296 (17)	163 (1)

Symmetry codes: (i) 
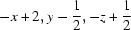
; (ii) 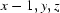.

**Fig. 1.**
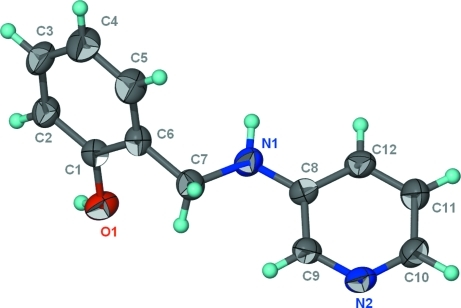
Thermal ellipsoid plot (Barbour, 2001) of C12H12N2O at the 50% probability level; hydrogen atoms are drawn as spheres of arbitrary radius.

## supplementary crystallographic information

### Comment

There are numerous studies on the Schiff bases derived by condensing salicyldehyde and an aromatic amine. In this study, the azomethine double-bond of *N*-salicylidene-3-aminopyridine (Csaszar, 1990; Kaya & Guelel, 2005; Robert *et al.* 2009) is reduced by sodium borohydride to yield the title secondary amine (Scheme I). The two aromatic rings at either ends of the –CH_2_–NH– link of C_12_H_12_N_2_O are twisted by 68.79 (7)° (Fig. 1). The hydroxy substituent is hydrogen-bond donor to the N atom of the pyridyl ring of an adjacent molecule, and the hydrogen bond generates a linear chain along the *b*-axis. It is also hydrogen-bond acceptor to the amino group of another adjacent molecule; the two hydrogen bonds lead to the formation of a layer structure (Table 1).

### Experimental

A solution of 3-aminopyridine (1 mmol) and salicylaldehyde (1 mmol) in toluene (50 ml) was heated for 10 h. The solvent was removed under vacuum, and the residue was reduced in absolute methanol by sodium borohydride. Colorless crystals were obtained by recrystallization from methanol; yield 80%.

### Refinement

Carbon-bound H-atoms were placed in calculated positions (C–H 0.93–0.97 Å) and were included in the refinement in the riding model approximation, with U_iso_(H) set to 1.2U_eq_(C). The amino and hydroxy H-atoms were located in a difference Fourier map, and were refined with distance restraints N–H 0.88±0.01 Å and O–H 0.84±0.01 Å; their temperature factors were refined.

### Crystal data

**Table:**

C_12_H_12_N_2_O	*F*(000) = 424
*M**_r_* = 200.24	*D*_x_ = 1.291 Mg m^−^^3^
Monoclinic, *P*2_1_/*c*	Mo *K*α radiation, λ = 0.71073 Å
Hall symbol: -P 2ybc	Cell parameters from 5377 reflections
*a* = 5.8386 (11) Å	θ = 3.0–27.5°
*b* = 13.399 (3) Å	µ = 0.08 mm^−^^1^
*c* = 13.169 (3) Å	*T* = 293 K
β = 90.519 (6)°	Prism, colorless
*V* = 1030.1 (4) Å^3^	0.21 × 0.12 × 0.12 mm
*Z* = 4	

### Data collection

**Table:**

Rigaku R-AXIS RAPID IP diffractometer	2358 independent reflections
Radiation source: fine-focus sealed tube	1879 reflections with *I* > 2σ(*I*)
graphite	*R*_int_ = 0.042
ω scan	θ_max_ = 27.5°, θ_min_ = 3.0°
Absorption correction: multi-scan (*ABSCOR*; Higashi, 1995)	*h* = −7→7
*T*_min_ = 0.983, *T*_max_ = 0.990	*k* = −17→17
9845 measured reflections	*l* = −17→16

### Refinement

**Table:**

Refinement on *F*^2^	Primary atom site location: structure-invariant direct methods
Least-squares matrix: full	Secondary atom site location: difference Fourier map
*R*[*F*^2^ > 2σ(*F*^2^)] = 0.047	Hydrogen site location: inferred from neighbouring sites
*wR*(*F*^2^) = 0.137	H atoms treated by a mixture of independent and constrained refinement
*S* = 1.08	*w* = 1/[σ^2^(*F*_o_^2^) + (0.0752*P*)^2^ + 0.0952*P*] where *P* = (*F*_o_^2^ + 2*F*_c_^2^)/3
2358 reflections	(Δ/σ)_max_ = 0.001
144 parameters	Δρ_max_ = 0.26 e Å^−^^3^
2 restraints	Δρ_min_ = −0.18 e Å^−^^3^

### Fractional atomic coordinates and isotropic or equivalent isotropic displacement parameters (Å^2^)

**Table:**

	*x*	*y*	*z*	*U*_iso_*/*U*_eq_	
O1	1.17862 (17)	0.30493 (7)	0.38420 (7)	0.0517 (3)	
H1O	1.249 (3)	0.2486 (9)	0.3790 (12)	0.073 (5)*	
N1	0.6391 (2)	0.42170 (9)	0.32008 (9)	0.0512 (3)	
H1N	0.534 (2)	0.3813 (10)	0.3444 (11)	0.059 (4)*	
N2	0.61025 (18)	0.63154 (8)	0.14353 (8)	0.0493 (3)	
C1	1.0374 (2)	0.30367 (9)	0.46539 (9)	0.0396 (3)	
C2	1.0673 (2)	0.23629 (11)	0.54458 (10)	0.0499 (3)	
H2	1.1882	0.1911	0.5429	0.060*	
C3	0.9202 (3)	0.23577 (13)	0.62516 (10)	0.0591 (4)	
H3	0.9407	0.1897	0.6773	0.071*	
C4	0.7428 (3)	0.30289 (14)	0.62928 (11)	0.0669 (5)	
H4	0.6418	0.3022	0.6835	0.080*	
C5	0.7166 (3)	0.37110 (12)	0.55208 (11)	0.0597 (4)	
H5	0.5982	0.4173	0.5557	0.072*	
C6	0.8605 (2)	0.37334 (9)	0.46929 (9)	0.0431 (3)	
C7	0.8270 (2)	0.44915 (10)	0.38681 (11)	0.0484 (3)	
H7A	0.7961	0.5138	0.4169	0.058*	
H7B	0.9664	0.4547	0.3477	0.058*	
C8	0.5640 (2)	0.48446 (9)	0.24474 (9)	0.0416 (3)	
C9	0.6833 (2)	0.57012 (10)	0.21638 (10)	0.0449 (3)	
H9	0.8200	0.5850	0.2499	0.054*	
C10	0.4163 (2)	0.61131 (11)	0.09441 (11)	0.0545 (4)	
H10	0.3659	0.6543	0.0434	0.065*	
C11	0.2877 (2)	0.52808 (12)	0.11717 (11)	0.0586 (4)	
H11	0.1522	0.5152	0.0819	0.070*	
C12	0.3611 (2)	0.46471 (11)	0.19197 (10)	0.0528 (4)	
H12	0.2755	0.4083	0.2077	0.063*	

### Atomic displacement parameters (Å^2^)

**Table:**

	*U*^11^	*U*^22^	*U*^33^	*U*^12^	*U*^13^	*U*^23^
O1	0.0596 (6)	0.0440 (5)	0.0517 (6)	0.0094 (4)	0.0165 (5)	0.0042 (4)
N1	0.0588 (7)	0.0428 (6)	0.0519 (6)	−0.0104 (5)	−0.0083 (5)	0.0094 (5)
N2	0.0515 (6)	0.0442 (6)	0.0522 (6)	−0.0019 (5)	0.0093 (5)	0.0090 (5)
C1	0.0424 (6)	0.0385 (6)	0.0380 (6)	0.0015 (5)	0.0016 (5)	−0.0046 (5)
C2	0.0494 (7)	0.0527 (8)	0.0476 (7)	0.0131 (6)	0.0022 (6)	0.0037 (6)
C3	0.0645 (9)	0.0721 (10)	0.0408 (7)	0.0146 (7)	0.0030 (6)	0.0107 (6)
C4	0.0663 (9)	0.0880 (12)	0.0467 (8)	0.0213 (8)	0.0168 (7)	0.0033 (7)
C5	0.0570 (8)	0.0670 (10)	0.0551 (8)	0.0241 (7)	0.0097 (7)	−0.0013 (7)
C6	0.0448 (6)	0.0413 (7)	0.0432 (6)	0.0053 (5)	−0.0032 (5)	−0.0040 (5)
C7	0.0465 (7)	0.0396 (7)	0.0589 (8)	0.0025 (5)	−0.0045 (6)	0.0019 (6)
C8	0.0473 (6)	0.0377 (6)	0.0400 (6)	−0.0011 (5)	0.0038 (5)	−0.0009 (5)
C9	0.0403 (6)	0.0434 (7)	0.0511 (7)	−0.0022 (5)	0.0045 (5)	0.0029 (5)
C10	0.0592 (8)	0.0578 (8)	0.0465 (7)	0.0034 (6)	0.0008 (6)	0.0120 (6)
C11	0.0558 (8)	0.0701 (10)	0.0498 (8)	−0.0095 (7)	−0.0094 (6)	0.0057 (7)
C12	0.0584 (8)	0.0518 (8)	0.0481 (7)	−0.0169 (6)	−0.0026 (6)	0.0037 (6)

### Geometric parameters (Å, °)

**Table:**

O1—C1	1.3562 (15)	C4—H4	0.9300
O1—H1O	0.86 (1)	C5—C6	1.383 (2)
N1—C8	1.3695 (16)	C5—H5	0.9300
N1—C7	1.4472 (17)	C6—C7	1.4986 (17)
N1—H1N	0.88 (1)	C7—H7A	0.9700
N2—C10	1.3269 (18)	C7—H7B	0.9700
N2—C9	1.3312 (16)	C8—C12	1.3936 (18)
C1—C2	1.3892 (18)	C8—C9	1.3953 (18)
C1—C6	1.3933 (17)	C9—H9	0.9300
C2—C3	1.371 (2)	C10—C11	1.379 (2)
C2—H2	0.9300	C10—H10	0.9300
C3—C4	1.373 (2)	C11—C12	1.366 (2)
C3—H3	0.9300	C11—H11	0.9300
C4—C5	1.374 (2)	C12—H12	0.9300
			
C1—O1—H1O	110.3 (12)	C1—C6—C7	121.29 (12)
C8—N1—C7	121.31 (11)	N1—C7—C6	111.17 (11)
C8—N1—H1N	114.9 (10)	N1—C7—H7A	109.4
C7—N1—H1N	117.6 (10)	C6—C7—H7A	109.4
C10—N2—C9	119.43 (11)	N1—C7—H7B	109.4
O1—C1—C2	121.78 (11)	C6—C7—H7B	109.4
O1—C1—C6	118.47 (11)	H7A—C7—H7B	108.0
C2—C1—C6	119.73 (12)	N1—C8—C12	120.65 (11)
C3—C2—C1	120.58 (12)	N1—C8—C9	122.81 (11)
C3—C2—H2	119.7	C12—C8—C9	116.54 (12)
C1—C2—H2	119.7	N2—C9—C8	122.96 (12)
C2—C3—C4	120.35 (13)	N2—C9—H9	118.5
C2—C3—H3	119.8	C8—C9—H9	118.5
C4—C3—H3	119.8	N2—C10—C11	121.55 (12)
C3—C4—C5	119.01 (14)	N2—C10—H10	119.2
C3—C4—H4	120.5	C11—C10—H10	119.2
C5—C4—H4	120.5	C12—C11—C10	119.44 (13)
C4—C5—C6	122.24 (13)	C12—C11—H11	120.3
C4—C5—H5	118.9	C10—C11—H11	120.3
C6—C5—H5	118.9	C11—C12—C8	120.07 (12)
C5—C6—C1	118.05 (12)	C11—C12—H12	120.0
C5—C6—C7	120.66 (11)	C8—C12—H12	120.0
			
O1—C1—C2—C3	−179.14 (13)	C5—C6—C7—N1	−77.50 (16)
C6—C1—C2—C3	1.9 (2)	C1—C6—C7—N1	102.91 (14)
C1—C2—C3—C4	−0.9 (2)	C7—N1—C8—C12	−169.54 (13)
C2—C3—C4—C5	−0.7 (3)	C7—N1—C8—C9	10.6 (2)
C3—C4—C5—C6	1.2 (3)	C10—N2—C9—C8	−0.4 (2)
C4—C5—C6—C1	−0.2 (2)	N1—C8—C9—N2	−179.66 (12)
C4—C5—C6—C7	−179.76 (14)	C12—C8—C9—N2	0.5 (2)
O1—C1—C6—C5	179.63 (12)	C9—N2—C10—C11	0.2 (2)
C2—C1—C6—C5	−1.41 (19)	N2—C10—C11—C12	0.0 (2)
O1—C1—C6—C7	−0.77 (17)	C10—C11—C12—C8	0.1 (2)
C2—C1—C6—C7	178.19 (11)	N1—C8—C12—C11	179.81 (14)
C8—N1—C7—C6	174.01 (12)	C9—C8—C12—C11	−0.3 (2)

### Hydrogen-bond geometry (Å, °)

**Table:**

*D*—H···*A*	*D*—H	H···*A*	*D*···*A*	*D*—H···*A*
O1—H1o···N2^i^	0.862 (14)	1.797 (14)	2.6568 (16)	175.0 (16)
N1—H1n···O1^ii^	0.881 (13)	2.376 (12)	3.2296 (17)	163.2 (12)

Symmetry codes: (i) −*x*+2, *y*−1/2, −*z*+1/2; (ii) *x*−1, *y*, *z*.
